# “I wanted to participate in my own care”: Evaluation of a Patient Navigation Program

**DOI:** 10.5811/westjem.2020.9.48105

**Published:** 2021-02-22

**Authors:** Elizabeth A. Samuels, Lauren Kelley, Timothy Pham, Jeremiah Cross, Juan Carmona, Peter Ellis, Darcey Cobbs-Lomax, Gail D’Onofrio, Roberta Capp

**Affiliations:** * Warren Alpert Medical School of Brown University, Department of Emergency Medicine, Providence, Rhode Island; † Project Access-New Haven, New Haven, Connecticut; ‡ Highland General Hospital, Department of Emergency Medicine, Oakland, California; § Yale University School of Medicine, Department of Medicine, New Haven, Connecticut; ¶ Yale University School of Medicine, Department of Emergency Medicine, New Haven, Connecticut; || University of Colorado School of Medicine, Department of Emergency Medicine, Aurora, Colorado

## Abstract

**Introduction:**

Patient navigation programs can help people overcome barriers to outpatient care. Patient experiences with these programs are not well understood. The goal of this study was to understand patient experiences and satisfaction with an emergency department (ED)-initiated patient navigation (ED-PN) intervention for US Medicaid-enrolled frequent ED users.

**Methods:**

We conducted a mixed-methods evaluation of patient experiences and satisfaction with an ED-PN program for patients who visited the ED more than four times in the prior year. Participants were Medicaid-enrolled, English- or Spanish-speaking, New Haven-CT residents over the age of 18. Pre-post ED-PN intervention surveys and post-ED-PN individual interviews were conducted. We analyzed baseline and follow-up survey responses as proportions of total responses. Interviews were coded by multiple readers, and interview themes were identified by consensus.

**Results:**

A total of 49 participants received ED-PN. Of those, 80% (39/49) completed the post-intervention survey. After receiving ED-PN, participants reported high satisfaction, fewer barriers to medical care, and increased confidence in their ability to coordinate and manage their medical care. Interviews were conducted until thematic saturation was reached. Four main themes emerged from 11 interviews: 1) PNs were perceived as effective navigators and advocates; 2) health-related social needs were frequent drivers of and barriers to healthcare; 3) primary care utilization depended on clinic accessibility and quality of relationships with providers and staff; and 4) the ED was viewed as providing convenient, comprehensive care for urgent needs.

**Conclusions:**

Medicaid-enrolled frequent ED users receiving ED-PN had high satisfaction and reported improved ability to manage their health conditions.

## INTRODUCTION

United States emergency department (ED) utilization has increased over the last three decades at a rate faster than the US population has grown.^1^,^2^ Frequent ED users, defined as individuals with four or more ED visits in a one-year period, comprise 4.5–8% of all ED patients, yet account for 21–28% of all annual ED visits.^3^ Frequent ED users are more likely to be older, have chronic illnesses, be Medicaid-insured, and have complex medical, behavioral health, and psychosocial needs.^3^–^7^ Approximately 85% of ED visits among Medicaid-enrolled frequent ED users result in discharge home. Many of these visits could occur in a primary care setting, which is more cost-effective and better for long-term patient outcomes.^8^–^10^ However, Medicaid patients have greater difficulty scheduling outpatient appointments compared to privately insured patients^11^ and encounter many barriers to accessing primary care, including lack of transportation and appointment availability.^6^,^8^,^12^–^14^

Patient navigation programs have been implemented across the US to help patients overcome barriers to accessing outpatient care.^15^–^17^ These programs provide services navigation, education, and care coordination.^8^ Many patient navigation programs have demonstrated success in reducing ED utilization and healthcare spending,^15^,^16^,^19^–^24^ but few have examined patient acceptability, experiences, and satisfaction. Evaluating patient experiences is critical for understanding which aspects of these programs successfully engage and meet patients’ needs. In this mixed-methods study, we evaluated patient perspectives, experiences, and satisfaction with an ED-initiated patient navigation (ED-PN) intervention for Medicaid-enrolled frequent ED users.^24^

## METHODS

### Study Setting and Population

We recruited participants from the Yale New Haven Hospital (YNHH) ED, a large, urban, academic hospital in New Haven, CT, treating over 100,000 adult patients annually. New Haven has over 130,000 residents (33% Black, 32% White, and 27% Hispanic). Of this population, 48% live at or below 200% of the federal poverty level.^25^ Twelve percent of Medicaid-enrolled YNHH ED patients are frequent ED users, accounting for 38% of all ED visits.^26^

### Participant Recruitment and Enrollment

Individuals were eligible for inclusion if they had the following characteristics: 18–62 years old; Medicaid-enrolled; English or Spanish speaking; residents of one of the twelve towns in the greater New Haven area; had 4–18 visits to a YNHH ED in the prior year; less than 50% of their prior year ED visits were for a psychiatric or substance use concern; and they were not being primarily treated for a psychiatric or substance use concern at the time of enrollment. We excluded from enrollment patients with frequent ED utilization for substance use disorders and behavioral health problems because they have additional and often complex clinical, behavioral, and social needs that the intervention was not designed or equipped to support.^24^

Participants were enrolled from March 2013–February 2014. After providing informed consent, they were randomized to either ED-PN or standard care using a previously generated, stratified randomization algorithm with a concealed sequence. Of the 100 individuals enrolled, 49 received the ED-PN intervention and 51 received standard care. The PNs were employed by Project Access-New Haven (PA-NH), a community-based non-profit that provides patient navigation to medical specialty services for people who are uninsured and Medicaid-enrolled.^27^ Details about study enrollment and randomization can be found in previously published manuscripts.^6^,^24^

**Population Health Research Capsule**

What do we already know about this issue?

Patient navigation (PN) programs provide services navigation, education, and care coordination, resulting in reduced ED use, hospitalizations, and healthcare costs.

What was the research question?

What are patient perspectives, experiences, and satisfaction with an ED-initiated PN program?

What was the major finding of the study?

Participants were highly satisfied with ED-initiated PN and reported increased self-confidence managing their health.

How does this improve population health?

EDs can use patient navigation programs to support and improve the health of marginalized and medically complex patients.

### Patient Navigation Intervention

Participants in the intervention arm received ED-PN for 12 months through PA-NH, a community-based nonprofit organization providing PN services for underserved Greater New Haven area residents.^27^ The navigation team included a bilingual (English/Spanish) patient nagivator and a nurse navigator. Both completed a two-day intensive training at the Harold Freeman Institute for Patient Navigation on how to provide PN and address barriers to care.^28^ Study navigators had supervision from a multidisciplinary team comprised of an emergency physician, a primary care physician, the PA-NH executive director, and a program coordinator. The study team met weekly, developed tailored plans for each participant, and provided direction on coordination of medical and social services.

The navigators scheduled post-ED primary care visits for each participant and offered accompaniment to up to three office visits. They met the participants prior to outpatient appointments to review their concerns and outline questions for the provider. Navigators encouraged participants to ask questions during the visit and helped create a post-visit task list based on the provider’s recommendations. The patient navigators also scheduled visits for provider-recommended specialty care and ancillary services.

Navigators contacted participants by phone every two weeks during weeks 0–4 and every four weeks during weeks 13–52 to review participants’ health and social needs. They also scheduled and reminded patients of medical appointments, addressed barriers to care, and provided referrals for social needs. Finally, navigators were available to answer participant questions and provide assistance as needed.

### Study Design

Participants completed a baseline questionnaire (via staff interview) at enrollment that included questions about demographics, health status/needs, healthcare utilization, and access/barriers to care. One-year post-enrollment and following completion of the ED-PN intervention, a research assistant not directly involved in PN conducted follow-up phone surveys to assess participant-reported healthcare utilization, access/barriers to care, and program satisfaction. Follow-up survey completion had no bearing on receipt of ED-PN. Survey questions included novel and validated questions to measure health literacy,^29^,^30^ healthcare utilization,^31^ health status,^32^ and self-efficacy for managing chronic diseases^33^ (Appendix A). Surveys were piloted with patient navigators for comprehension and lasted approximately 15–30 minutes. Responses were collected using a web-based platform (Qualtrics XM, Provo, UT). Respondents received a $25 gift card for participation.

Upon completion of the follow-up survey, English-speaking ED-PN participants were invited to participate in a qualitative interview about the PN program. The study team developed the interview guide, which was reviewed by patient navigators for understandability and iteratively revised (Appendix B). The interviewer had not previously interacted with any of the participants. Audiorecorded interviews were approximately 45–60 minutes in length and transcribed verbatim. Interviews were conducted until thematic saturation was reached. Participants received a $50 gift card for completing the interview. Interview and follow-up survey completion occurred following completion of ED-PN intervention. Participants were informed that participation in these assessments had no bearing on current or future services received. This study was approved by the Yale University Institutional Review Board.

## ANALYSIS

### Patient Surveys

We analyzed baseline and follow-up survey responses as proportions of total responses. The datasets analysed are available from the corresponding author on reasonable request.

### Patient Interview

The coding structure and categories followed the topical framework of the interview guide and were iteratively refined through group discussion. The coding classification scheme was finalized by consensus and applied to each transcript by at least two independent reviewers. Any coding discrepancies or ambiguities were resolved through discussion. Codes were applied to each transcript using ATLAS.ti version 5.2 (ATLAS.ti, Berlin, Germany). The study team reached consensus on a final thematic framework and identified illustrative quotes that represented the responses relevant to each theme.

## RESULTS

### Survey Results

Forty-nine participants received ED-PN. Over half were female (67%), nearly half (47%) were Black, most spoke English (86%), and over half (57%) worked at least part time (Table 1). Over half (65%) reported fair to poor health at baseline and most (86%) had at least one chronic health condition. At baseline, 48% reported not being able to get outpatient appointments as soon as needed and 70% reported receiving most of their healthcare in the ED (Table 2). Of the ED-PN participants, 80% (39/49) completed the post ED-PN survey (Table 3). After receiving ED-PN, participants were more likely to report “usually” or “always” getting medical appointments as soon as needed (94% vs 53%) and having their medical questions answered the same day (96% vs 50%). Participants also reported decreased use of the ED as their primary site of care (30% vs.70%), fewer barriers to care, and increased confidence in their ability to coordinate their own care and self-manage their medical conditions (Table 3).

Participants reported high overall satisfaction and identified assistance with scheduling appointments, appointment reminders, follow-up calls, and having someone to talk to about their health as the most helpful navigation services (Figure, Panels A and B). Participant reported satisfaction with ED-PN services was high. All participants reported being overall satisfied with ED-PN, and 89.7% (35/39) reported being very satisfied. The majority (87.2%, 34/39) also reported being very satisfied with how long they had to wait for a medical appointment. Most (87.2%, 34/39) reported that it was easy to follow treatment advice after getting ED-PN and easy to get care (76.9%, 30/39). After receiving ED-PN, most thought their ability to get care had improved (84.6%, 33/39).

### Interview Results

We conducted 11 interviews. Compared to the ED-PN group, most interviewed participants (n = 11) were female (82% vs 67%) and Black (55% vs 47%) and fewer had full-time employment (9% vs 18%). Interviewees were otherwise similar to the overall ED-PN group in their sociodemographic characteristics and reported health (Tables 1 and 2). Four main themes emerged: 1) Patient nagivators were perceived as effective healthcare coordinators and advocates who provided continuity and individualized support (Theme 1); 2) health-related social needs were frequent drivers of and barriers to healthcare utilization that required PN assistance (Theme 2); 3) primary care utilization depended on clinic accessibility and quality of interpersonal relationships with providers and staff (Theme 3); and 4) participants characterized the ED as providing convenient, comprehensive care for urgent needs and filling gaps in primary care access (Theme 4)(see Supplemental Table).

Theme 1: Patient navigators perceived as effective healthcare coordinators and patient advocates who provided continuity and individualized support.

Participants provided unanimously positive feedback about PN support. Many described feeling relieved about finally receiving the assistance they felt they needed. One participant observed, “You feel like nobody elsewhere is helping you and they’re there to help… I was at my wit’s end when [the PN] came to me. I was so fed up.” (Participant 5)

Strong PN-patient relationships were consistently cited as a key program component. One participant described their relationship with the patient navigator as, “Wonderful… I felt that they cared, they really cared, not just about me, but actually me.” (Participant 10) Participants linked this directly to the development of self-worth and trust. PN services were viewed as non-judgmental, unconditional, and made participants feel comfortable. “They made me feel like, ‘This is my hand extended out to you, whether you want it or you don’t.’ They didn’t make me feel bad, they made me feel comfortable.” (Participant 8)

Patient navigators also educated participants about healthcare utilization and what to expect from healthcare visits. Some participants said this allowed them to “[Know] my rights a little bit more.” (Participant 6) Drawing from PN education and support, participants described developing improved self-efficacy navigating the healthcare system. One participant reported feeling, “More comfortable to go back to my primary care doctor and say ‘Hey, you’re my primary care doctor, you’re supposed to be the one to see me and give me care’… I felt stronger… I felt empowered to make an appointment.” (Participant 6) The participant continued: “Within a year, I was able to…go to the primary care doctor, go to the dentist. I was able to get going, I became familiar. I was driven, I … wanted to participate in my own care.” (Participant 6)

Participants described developing very strong bonds and trust with their patient navigators and indicated that they made a noticeable difference in their lives. One participant described, “If it wasn’t for [the PN] I’m telling you; I wouldn’t have been at none of these appointments. If it wasn’t for [the PN] checking on me, calling me, asking ‘did you do this, did you do that,’ I really was lost.” (Participant 8)

Theme 2: Navigators helped patients address health-related social needs that were drivers and barriers to healthcare utilization.

Social, economic, and personal considerations were common factors that impacted participants’ healthcare utilization. Several participants commented that navigators helped them prioritize their health and healthcare appointments despite social barriers and competing concerns. One noted, “[The PNs] helped out because when I have so many things on my mind, like … my daughter and her homework, or me trying to find the, not the right job, but the most beneficial employment... Is there food in the house, does she have the right shoes, this and that…So, for you to call me and remind me [to go to my appointments], that’s a beautiful thing.” (Participant 4)

Transportation, caregiver responsibilities, and housing were commonly cited barriers to accessing primary care. Patient navigators frequently assisted with transportation. One individual noted: “for bus passes you got to call seven days before and sometimes it’d come the day after my appointment, but if I called [the PN], they’d get right on that phone, call transportation and they’d send me a taxi that morning for my appointment.” (Participant 10)

A number of participants experienced major life events, such as incarceration of family members or family health problems, that impacted their health, further demonstrating that additional support is be needed beyond the scope of the PN program. In such situations, navigators directed patients to local resources and provided emotional support. While most participants did not report receiving assistance with health-related social needs (Figure, Panel C), participants who did use these services reported positive experiences. However, the ED-PN intervention was not designed for comprehensive navigation to address these needs. During the course of the study, staff often noted feeling limited in their ability to address health-related social needs, particularly housing.

Theme 3: Primary care utilization was driven by clinic accessibility and quality of interpersonal relationships

Appointment availability, interactions with clinic providers and staff, and perceived care quality, thoroughness, and continuity were commonly mentioned factors impacting primary care utilization. Provider continuity and familiarity with one’s past medical history cultivated trust and comfort. However, many participants who received care at the primary care resident clinic connected their decreased clinic utilization to their dissatisfaction with the clinic stemming from lack of trust in providers, feeling rushed during appointments, and lack of confidence in the quality of clinic care.

The high volume of patients at the primary care clinic and perceived lack of organization were viewed as compromising patient care. Explained one participant, “With the primary care clinic, is for one they are overpopulated. They’re not able to assess each patient the way that they should…It’s always hectic… when you walk into the clinic, you can just feel the energy of people waiting for two and three hours to be seen by a doctor. It’s no organization in the waiting room. It’s a mess. The clinic is a mess.” (Participant 6)

Participants were also frustrated with lack of provider consistency at the primary care resident clinic. One participant explained, “You don’t want to keep seeing different people. You want to see the same person…You’re always bounced around to different people where you’d have to explain your whole story to because they don’t know you. So, there goes your 15 minutes right there.” (Participant 5)

Several patients commented that PN accompaniment to primary care provider visit(s) was beneficial and improved their overall experience of care. One noted: “[The PN] helped me realize you’re paying for this; you have the right to ask questions… and that helped me out a lot.” (Participant 8) Another participant described being treated differently when the PN attended her appointment: “They were all so nice, never happened before… I don’t know if they’re intimidated... because she was a woman with a badge, dressed up nice, paperwork folders... I was treated perfect.” (Participant 5)

Theme 4: Emergency department provides convenient, comprehensive care for urgent needs and fills gaps in primary care access.

Most participants used the ED to fill gaps in primary care and described the ED as a convenient place to obtain comprehensive care for urgent needs. When weighing options for where to seek care, patients frequently viewed the ED as the only available option for urgent needs. Said one participant, “I just said, ‘Forget about [making an appointment].’ I couldn’t take the pain anymore. So, I ended up in the emergency room.” (Participant 1)

Illness acuity in combination with other issues, particularly limited transportation, also brought people to use the ED. Inability to get a timely appointment was frequently mentioned as a reason to use the ED. “When I tried to call the primary care center, they weren’t available the way I needed them to be available,” said one participant. She continued, “If I felt there was something important and medically urgent and to them it wasn’t, I wanted it that same day and they would do three, four days later and I felt to myself it was important, I would just go straight to the ED.” (Participant 5) Waiting to be seen in the ED was not viewed positively, but not necessarily a deterrent given perceived or actual inability to get timely primary care appointments. Described one participant, “It’s normal to be a long wait [at the ED]. I don’t bash that. Sometimes it’s agitating but there are so many people like me out there that can’t get help at primary care doctors and physicians that they get so packed.” (Participant 5)

After the program, several participants recognized the benefits of using primary care for comprehensive care and the ED for discrete problems. One participant described, “If you go to the primary care it’s like you’re having an appointment, they can check everything that you think could possibly be wrong with you at this point in time. But when you go to the ER, you’re treated for whatever you came there for. Like I broke my foot, but I have a cough, they’re gonna treat your foot, but not the cough.” (Participant 12)

Some participants reported continuing to use the ED after the ED-PN intervention when they were acutely ill, unable to get a primary care appointment, or due to hours of operation. A few participants noted that they preferred the convenience and perceived comprehensiveness of ED care. They also acknowledged that being seen in the ED could also expedite access to outpatient care. One participant described, “And you know, [ED providers] will get things going… I know that once I get in the back, once I tell them what is going on, they will do a CT scan, they will do x-rays, they will do all the emergencies that could be going on with me and refer me to my doctor and then I’ll get an appointment to my primary care sooner.” (Participant 6)

## DISCUSSION

In this mixed methods evaluation, Medicaid-enrolled frequent ED users were highly satisfied with the ED-PN intervention and reported increased healthcare access and self-confidence in managing their health conditions. Our findings underscore the value of navigation services to patients beyond traditional healthcare utilization and cost metrics. Participants in our study described many social factors that affected their ability to attain and maintain adequate health and access to primary care including transportation, difficulty scheduling time off from work, and problems with insurance. Given the importance and frequency of these factors in people’s lives and their impact on healthcare utilization, future navigator programs need to prioritize addressing unmet social needs, help that is not traditionally given in the healthcare system. Participants noted that they needed additional help with health-related social needs, and staff reported feeling limited in their ability to address these issues. Further studies are needed to understand how best to assess and address health related social needs and to identify needs specific to different patient groups, particularly people who do not speak English and were not included in study interviews.

Key factors driving decisions of where to seek healthcare included quality of relationships with primary care providers, appointment availability, and time spent with providers. Participants reported a significant decrease in ED utilization, which is consistent with objective findings from prior program evaluations that demonstrated reduced ED utilization and hospitalizations among people receiving PN and overall cost savings for participants who were older and had lower health literacy.^24^ Despite these changes, several participants felt they had better relationships with the ED, where their history was readily accessible in the electronic health record and they would spend several more hours at a time interacting with caregivers, than with their primary care offices.

There was an overwhelming perception that the ratio of time spent making and waiting for the appointment vs time spent in the appointment was out of proportion. In the ED, on the other hand, despite long wait times, patients felt assured that they would receive a thorough workup. In addition, once in the ED, they were able to receive additional services without delay (eg, specialty consults, diagnostic tests) rather than making future appointments that might require long wait times for appointments, transportation challenges, time off from work, and childcare issues. These findings are consistent with previous studies that have evaluated the impact of these factors, often referred to as opportunity costs on healthcare utilization.^34^ The ED with 24/7/365 day availability is a convenient site of care that people can access when these costs (time off work, childcare, transportation) can be minimized. This further underscores the need for a patient-centered health system that lowers barriers to preventative and primary care by minimizing patients’ opportunity costs when accessing healthcare.^17^,^34^

## LIMITATIONS

This study has several limitations. Study participants were Medicaid-enrollees residing in and aroundNew Haven, CT and may have had specific needs not necessarily generalizable to different populations, rural areas, or smaller EDs. However, this study was designed for hypothesis generation regarding patient perspectives on PN programs. Interviewees varied slightly by gender, race, and employment status compared to non-interviewees and may have had different degrees of unmet social needs compared to the larger intervention group. Due to the small study sample and the fact that interview participation was optional, results may be subject to selection bias resulting in an increase in positive reported experiences with the PN program. Additionally, we did not interview those patients from the study control arm who primarily spoke Spanish, or those we could not reach by phone after study completion; these participants may have expressed different views. However, the types of barriers that interviews described, and the four thematic domains that emerged are comparable to findings from similar research.^14^

## CONCLUSION

This study provides a deeper understanding of patient-oriented outcomes associated with patient navigation programs in addition to traditional metrics evaluated by other programs.^17^ Our findings suggest additional factors – the relationship between the navigator and clients, having a person in the healthcare system whom participants felt they could rely on and trust, and addressing health-related social needs – were highly valued by participants. This further supports the importance of tailoring navigation services to each individual. While improved healthcare utilization and patient satisfaction are important outcomes, future investigations are needed to understand how to optimize navigation programs to provide sustained support over time and improve self-reported health and quality of life. Future cost analyses of patient navigation programs that take into account program cost and changes in hospitalizations and medical complications can further assess the value of these programs.

## Supplementary Information







## Figures and Tables

**Figure:**
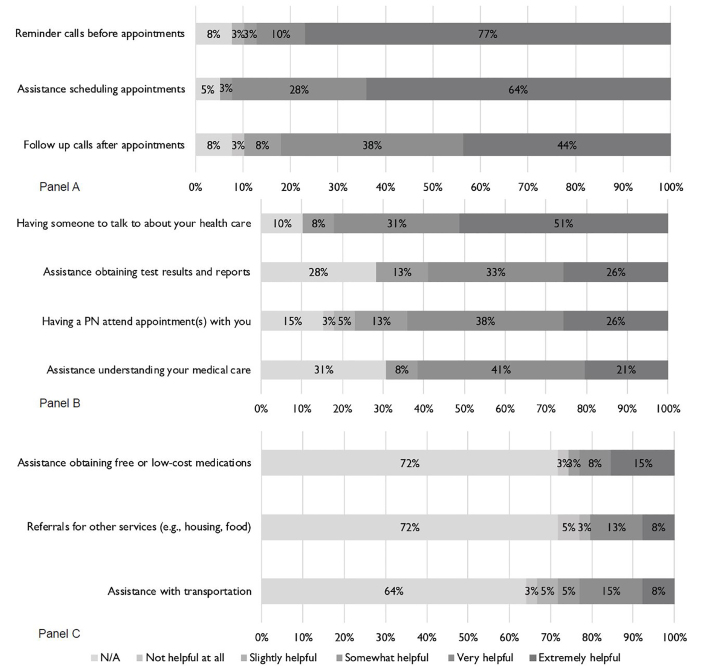
Helpfulness of Navigation Components. Participant reported helpfulness of patient navigation services including assistance with appointment scheduling and reminder calls (Panel A), health system navigation (Panel B), and health-related social needs (Panel C). Responses are reported as proportions of total responses in categories of NA, Not helpful at all, Slightly Helpful, Somewhat Helpful, Very Helpful, and Extremely Helpful. *PN*, Patient navigator.

**Table 1:** Demographics of all participants receiving emergency department patient navigation and individuals interviewed.

	All navigation recipients (n=49) N(%)	Interviewees (n=11) N(%)
Gender		
Female	33 (67)	9 (82)
Age (mean years)	40.2	37.1
Race/ethnicity		
Hispanic/Latino	19 (39)	3 (27)
Non-Hispanic, Black	23 (47)	6 (55)
Non-Hispanic, White	5 (10)	1 (9)
Non-Hispanic, American Indian/Alaska Native	1 (2)	1 (9)
Non-Hispanic, Other	1 (2)	0 (0)
Primary language		
English	42 (86)	10 (91)
Spanish	7 (14)	1 (9)
Marital status		
Never married	21 (43)	5 (45)
Married/civil union/living with partner	12 (24)	2 (18)
Separated/divorced/widowed	16 (33)	4 (36)
Education		
Elementary/grade school	5 (10)	0 (0)
Some high school	6 (12)	1 (9)
High school/GED	18 (37)	5 (45)
Some college (no degree)	14 (29)	4 (36)
Associate’s/Bachelor’s Degree	6 (12)	1 (9)

*GED*, General Educational Development exam.

**Table 2:** Social, economic, and health characteristics of participants receiving emergency department patient navigation and individuals interviewed.

	All navigation recipients (n=49) N (%)	Interviewees (n=11) N (%)
Food insecurity (not enough food/money to buy food in past 30 days)		
Never	21 (43)	4 (36)
Sometimes	21 (43)	6 (55)
Often	7 (14)	1 (9)
Housing instability		
Did not spend last 7 days in own place	10 (20)	3 (27)
Homeless in past year (≥1×)	6 (12)	1 (9)
Health literacy		
Mean REALM score (scale: 0–7)	5.0	5.2
Low health literacy (REALM score <=6), N(%)	33 (67)	6 (55)
Health status (self-report)		
Poor	11 (22)	3 (27)
Fair	21 (43)	5 (45)
Good	9 (18)	2 (18)
Very good	4 (8)	0 (0)
Excellent	4 (8)	1 (9)
Healthy days measure (mean days)		
Poor physical or mental health (N days in last 30 days)	21.0	19.2
Unable to do usual daily activities (N days in last days)	11.5	13.2
Chronic conditions (self-reported)		
Hypertension	21 (43)	4 (36)
High cholesterol	11 (22)	2 (18)
Coronary heart disease	3 (6)	1 (9)
Congestive heart failure	3 (6)	1 (9)
Heart attack	2 (4)	1 (9)
Asthma	22 (45)	4 (36)
Diabetes	14 (29)	2 (18)
Chronic lung disease/COPD	2 (4)	1 (9)
Depression	27 (55)	5 (45)
Anxiety	22 (45)	5 (45)
Other mental illness	5 (10)	2 (18)
Cancer	3 (6)	0 (0)
Stroke	2 (4)	1 (9)
At least one of the above chronic conditions	42 (86)	8 (73)

*REALM*, Rapid Estimate of Adult Literacy in Medicine; *COPD*, chronic obstructive pulmonary disease.

**Table 3:** Participant-reported ability to get appointments and answers to medical questions, barriers to care, and ability to coordinate and manage their medical conditions before and after receiving emergency department patient navigation.

	All Navigation Recipients (n=49)		Interviewees* (n=11)	
	
	PRE (n=49) N (%)	POST (n=39) N (%)	PRE (n=11) N (%)	POST (n=9) N (%)
Appointments as soon as needed (past 12 months)				
Never	10 (24)	1 (3)	5 (45)	0 (0)
Sometimes	10 (24)	1 (3)	2 (18)	0 (0)
Usually	7 (17)	4 (11)	0 (0)	1 (11)
Always	15 (36)	30 (83)	4 (36)	8 (89)
Medical questions answered same day, regular business hours (past 12 months)				
Never	9 (26)	0 (0)	3 (30)	0 (0)
Sometimes	8 (24)	1 (4)	3 (30)	0 (0)
Usually	5 (15)	3 (13)	1 (10)	0 (0)
Always	12 (35)	19 (83)	3 (30)	8 (100)
Barriers to care				
Cost	20 (41)	5 (13)	1 (9)	1 (11)
Transportation	32 (65)	16 (41)	9 (82)	5 (56)
Work schedule	12 (24)	6 (15)	2 (18)	3 (33)
Childcare	9 (18)	4 (10)	2 (18)	1 (11)
Unsure where/how to get care	20 (41)	3 (8)	4 (36)	1 (11)
Hard to find Medicaid providers	18 (37)	8 (21)	6 (55)	3 (33)
Difficulty getting appointments soon enough	28 (57)	10 (26)	5 (45)	2 (22)
Difficulty communicating with providers	6 (12)	1 (3)	2 (18)	1 (11)
Difficulty understanding medical infor-mation	17 (35)	2 (5)	4 (36)	1 (11)
Difficulty filling prescription medications	10 (20)	3 (8)	2 (18)	1 (11)
Unhappy with past experience with provider	17 (35)	7 (18)	5 (45)	2 (22)
Prefer to treat self	12 (24)	0 (0)	1 (9)	0 (0)
Disability	8 (16)	1 (3)	2 (18)	1 (11)
None	3 (6)	10 (26)	0 (0)	1 (11)
Prepared to coordinate own care				
Not at all prepared	10 (20)	2 (5)	3 (27)	2 (22)
Mostly not prepared	5 (10)	5 (13)	2 (18)	0 (0)
Somewhat prepared	19 (39)	16 (41)	4 (36)	6 (67)
Very prepared	15 (31)	16 (41)	2 (18)	1 (11)
Confidence in Self-Management of Medical Condition(s) (1=Not at all confident – 10 = Totally confident)				
Mean	6.61	7.74	6.00	6.78

* All interviews occurred after receiving emergency department patient navigation. Results here are interviewee responses to the pre- and post-survey conducted before and after receiving emergency department patient navigation.
