# Supplementary material for: “I wanted to participate in my own care”: Evaluation of a Patient Navigation Program
Source: West J Emerg Med. 2021 Feb 22;22(2):417–26. doi: 10.5811/westjem.2020.9.48105 (PMC7972383; doi:10.5811/westjem.2020.9.48105)
Supplement: Supplementary file 1 [file wjem-22-417-s001.docx]

**PA-NH Urgent Specialty Care Program**

**Follow-Up Survey 2014**

PA-NH ID: _________________________

Date: _________________________

Interviewer: _________________________

Is this survey being conducted in: 🞎 English 🞎 Spanish

Is this patient currently enrolled in PA-NH (i.e., active status)? 🞎 Yes 🞎 No [uninsured patients only]

**INTRODUCTION – NAVIGATED UNINSURED PATIENTS**

Hello.  My name is __________ and I am calling from Project Access-New Haven.

I am calling to see how you are doing and to ask some questions about your health, the services you received through Project Access, and your overall experience with the program and health care system.

It is very important for us to hear back from people who participate in Project Access.  Your feedback will help us to learn about the services you received and find ways that we may improve the program.  It will also help make sure that we can continue to provide services to those who need it in the future.

I have a brief survey that will take about 30 minutes to complete.  All information will be kept confidential – meaning we will not use your name or share your individual responses with anyone outside of Project Access. To thank you for your time and feedback, we are offering a $20 gift card to a local store if you complete the survey.

Is it ok to go ahead with the survey? If not, is there a better time to reach you?

*Hola. Mi nombre es ____________ y estoy llamando de Project Access de New Haven.*

*Lo/la estoy llamando para saber sobre su experiencia con Project Access, el estado de su salud, y su experiencia en general con el sistema de ayuda médica.*

*Es muy importante para nosotros escuchar sobre la experiencia de las personas que han participado de Project Access. Su experiencia nos ayudara a aprender sobre los servicios que usted ha recibido y para encontrar nuevas maneras de mejorar el programa. También nos ayudara a asegurarnos que podamos continuar a proveer los servicios a usted y a otros que necesiten de ayuda en el futuro.*

*Tengo un corto cuestionario que tomara 30 minutos de completar. Toda la información será guardada en forma confidencial-lo que quiere decir que no compartiremos sus respuestas con nadie afuera de Project Access. Para agradecerle por su tiempo y su opinión, le estamos ofreciendo una tarjeta de regalo de $20 de una tienda local, si usted completa la encuesta.*

*¿Estaría bien si continuamos y comenzamos con el cuestionario? Si no, ¿hay otro momento más oportuno para que nos contactemos con usted?*

**INTRODUCTION – NAVIGATED FREQUENT ED USER PILOT PATIENTS**

Hello.  My name is __________ - I am calling from Project Access-New Haven.

I am calling because you have been participating in a program where you received assistance with your health care through Project Access. It has been about one year since you enrolled, so I would like to ask you some final questions about your health and experiences with the program and health care system. The information you provide will be used to help improve health care services for patients like you.

I have a brief survey that will take about 30 minutes to complete. All information will be kept confidential – meaning we will not use your name or share your individual responses with anyone outside of Project Access. To thank you for your time and feedback, we are offering a $25 gift card to a local store if you complete the survey.

Is it ok to go ahead with the survey? If not, is there a better time to reach you?

*Hola. Mi nombre es______________. Lo/La estoy llamando de Project Access-New Haven.*

*Lo/La estoy llamando porque usted ha estado participando de un programa donde usted recibió asistencia con su cuidado médico a travez de Project Access. Ha pasado ya casi un año desde que usted comenzó en el programa, por eso me gustaría hacerle algunas preguntas finales acerca de su salud y su experiencia con el programa y el sistema de salud médica. La información que usted provee será usada para ayudarnos a mejorar el sistema médico para pacientes como usted.*

*Tengo un corto cuestionario que tomara 30 minutos de completar. Toda la información será guardada en forma confidencial-lo que quiere decir que no usaremos su nombre o sus respuestas individuales con nadie afuera de Project Access. Para agradecerle por su tiempo y su opinión, le estamos ofreciendo una tarjeta de regalo de $25 de una tienda local, si usted completa la encuesta.*

*¿Estaría bien si continuamos y comenzamos con el cuestionario? Si no, ¿hay otro momento más oportuno para que nos contactemos con usted?*

**INTRODUCTION – CONTROL FREQUENT ED USER PILOT PATIENTS**

Hello.  My name is __________ - I am calling from Project Access-New Haven.

I am calling because you agreed to participate in a research study when you were in the Yale-New Haven Hospital Emergency Room about a year ago. I would like to check in to see how you are doing and ask some questions about your health and experiences with the health care system since that visit. The information you provide will be used to help improve health care services for patients like you.

I have a brief survey that will take about 30 minutes to complete. All information will be kept confidential – meaning we will not use your name or share your individual responses with anyone outside of Project Access. To thank you for your time and feedback, we are offering a $25 gift card to a local store if you complete the survey.

Is it ok to go ahead with the survey? If not, is there a better time to reach you?

*Hola. Mi nombre es______________. Lo/La estoy llamando de Project Access-New Haven.*

*Lo/La estoy llamando porque usted estuvo de acuerdo en participar de un estudio de investigación cuando usted se encontraba en la Sala de Emergencias del Hospital de Yale-New Haven como hace un año atrás. Me gustaría hablar con usted para saber cómo está usted ahora y hacerle algunas preguntas acerca de su salud y su experiencia con el sistema de salud médica desde esa visita. La información que usted nos provea será usada para mejorar el sistema de servicios médicos para pacientes como usted.*

*Tengo un corto cuestionario que tomara 30 minutos de completar. Toda la información será guardada en forma confidencial-lo que quiere decir que no usaremos su nombre o sus respuestas individuales con nadie afuera de Project Access. Para agradecerle por su tiempo y su opinión, le estamos ofreciendo una tarjeta de regalo de $25 de una tienda local, si usted completa la encuesta.*

*¿Estaría bien si continuamos y comenzamos con el cuestionario? Si no, ¿hay otro momento más oportuno para que nos contactemos con usted?*

**DEMOGRAPHIC CHARACTERISTICS**

**The first few questions are about your current insurance and employment:**

***Las primeras preguntas son acerca de su seguro médico actual y su empleo:***

Do you have any kind of health care coverage, including health insurance, prepaid plans such as HMOs, or government plans such as Medicaid, Medicare, or Indian Health Service? [Adapted from BRFSS]

***¿****Tiene algún tipo de cobertura de salud, incluyendo seguro de salud, planes prepagados como HMO o planes de gobiernos como Medicaid, Medicare o Servicios de Salud Indio?*

🞎 Yes

*Sí*

🞎 No

*No*

If yes:

What type? Check all that apply. [PA-NH Baseline]

***¿***Qu*é* tipo? Marque todas las que apliquen.

🞎 Medicaid/HUSKY

*Medicaid/HUSKY*

🞎 Medicare

*Medicare*

🞎 Private insurance through Access Health CT

*Seguro privado a travez de Access Health CT*

🞎 Private insurance through an employer

*Seguro privado a travez de un empleador*

🞎 Private insurance not through Access Health CT or an employer

*Seguro privado no a travez de Access Health CT o de un empleador*

🞎 Other (specify): ______________________________

*Otro (especifique):* ______________________________

What is your current employment status? [Various sources]

***¿****Cuál es su situación de empleo?*

🞎 Employed full-time

*Empleado tiempo completo*

🞎 Employed part-time

*Empleado parte del tiempo*

🞎 Unemployed and looking for work
 *Desempleado y buscando empleo*

🞎 Unemployed, but not currently looking for work (e.g., student, homemaker, retired)
 *Desempleado pero no está buscando empleo/trabajo*

🞎 Unable to work (e.g., health concerns, disability)

*Incapaz de trabajar*

**HEALTH STATUS**

**Now I am going to ask about your health:**

***Ahora le voy a preguntar algunas preguntas acerca de su salud:***

Would you say that in general your health is: [CDC Healthy Days]

*¿Diría usted que en general su salud es:*

🞎 Excellent

*Excelente*

🞎 Very good

*Muy Buena*

🞎 Good

*Buena*

🞎 Fair

*Regular o*

🞎 Poor

*Mala*

Now thinking about your physical health, which includes physical illness and injury, for how many days during the past 30 days was your physical health not good? [CDC Healthy Days]

*Ahora refiriéndonos a su salud física, la cual incluye enfermedades físicas y accidentes:*

*¿Cuántos días, durante los últimos treinta días, su salud no fue tan buena?,*

___ / 30

Now thinking about your mental health, which includes stress, depression, and problems with emotions, for how many days during the past 30 days was your mental health not good? [CDC Healthy Days]

*Ahora refiriéndonos a su salud mental, la cual incluye tensión, depresión y problemas emocionales:*

*¿Cuántos días, durante los últimos treinta días, su salud no fue tan buena?*

___ / 30

During the past 30 days, for about how many days did poor physical or mental health keep you from doing your usual activities, such as self-care, work or recreation? [CDC Healthy Days]

*Durante los últimos treinta días, ¿por cuántos días el mal estado de salud física o mental le impidió realizar sus actividades habituales, tales como cuidado personal, trabajo o recreación?*

___ / 30

**HEALTHCARE UTILIZATION & EXPERIENCES**

**Now I am going to ask about your doctors and health care providers:**

***Ahora le voy a preguntar acerca de sus médicos y proveedores de atención medica:***

Is there a place that you usually go when you are sick or need advice about your health?

[NHANES/MEPS/etc.]

***¿****Hay un lugar que usted suele ir cuando usted está enfermo o necesita consejo sobre su salud?*

🞎 Yes

*Sí*

🞎 There is no place

*No hay ningún lugar*

🞎 There is more than one place

*Hay mas de un lugar*

If “yes” or “there is more than one place”:

What kind of place do you go to most often: is it a clinic, doctor's office, emergency room, or some other place? [NHANES/MEPS]

***¿****A qué tipo de lugar usted va la mayoría de las veces?* ***¿****A una clínica, oficina de doctor, sala de emergencia, u otro lugar?*

🞎 Clinic or health center (e.g., Fair Haven, Hill)

*Clínica o Centro de salud*

🞎 Doctor’s office or HMO (e.g., private office or multispecialty practice)

*Oficina privada de doctor o HMO*

🞎 Hospital emergency room

*Sala de emergencia de hospital*

🞎 Hospital outpatient department (e.g., Primary Care Center at YNHH or YNHH SRC)

*Clínica de pacientes externos del hospital*

🞎 Some other place (specify): ________________________________________

*Otro lugar (especifique):__________________________________________*

🞎 Don’t know

*No sabe*

How long have you been seeing this doctor/care provider? [Erica’s Measure]

***¿****Qué tiempo hace que está viendo a este doctor/proveedor?*

🞎 Less than 2 years

*Menos de 2 años*

🞎 2 years or more

*2 años o mas*

How well does that doctor/care provider know you and your medical history? [Erica’s Measure]

***¿****Qué tan bien ese doctor/proveedor le conoce a usted y a su historial médico?*

🞎 Very Well

*Muy bien*

🞎 Somewhat

*Más o menos*

🞎 Not at all

*Para nada bien*

Is your doctor/care provider or his/her staff good at following up with you on appointments and test results? [Erica’s Measure]

***¿****Son buenos su médico/personal de atención médica o asistentes en el seguimiento de sus citas y resultados de sus exámenes?*

🞎 Very Well

*Muy buenos*

🞎 Somewhat

*Más o menos*

🞎 Not at all

*No en absoluto*

Do you have one person who you think of as your personal/primary doctor or health care provider? A personal/primary doctor or provider is the person you would see if you need a checkup, want advice about a health problem, or get hurt or sick. Do not include specialists. [Adapted from BRFSS/CAHPS]

***¿****Tiene usted a una persona a quien usted considera su médico de cabecera? Un médico de cabecera es le persone que usted ve si necesite un físico, necesite un consejo sobre algún problema de salud, o se lastima o esta enfermo. No incluya a especialistas.*

🞎 Yes

*Sí*

🞎 No

*No*

During the past 12 months, how many times have you seen a doctor or health care provider about your health at a doctor's office, clinic, at home or some other place? Do not include times you were seen in the ED or hospitalized overnight. [Adapted from NHANES]

*En los últimos 12 meses, ¿cuántas veces ha visto a un médico o proveedor de atención medica acerca de su salud en un consultorio privado, clínica, en su casa o en otro lugar? No incluya las veces que visito la sala de Emergencia o fue hospitalizado durante la noche.*

____

**Now I am going to ask about Emergency Room visits and hospitalizations:**

***Ahora le voy a preguntar sobre visitas a la Sala de Emergencia y hospitalizaciones durante la noche:***

During the past 12 months, were you a patient in a hospital overnight? Do not include an overnight stay in the emergency room. [NHANES]

*En los últimos 12 meses, ¿fue internado en el hospital durante la noche? No incluya si ha pasado una noche en la sale de emergencia.*

🞎 Yes

*Sí*

🞎 No

*No*

If yes:

How many different times did you stay in any hospital overnight or longer during the past 12 months? Do not count total number of nights, just total number of hospital admissions for stays that lasted 1 or more nights. [NHANES]

*Durante los últimos 12 meses, ¿cuántas veces se ha quedado en el hospital durante una noche o más? No cuente el total de las noches en el hospital, solo el total de admisiones que duraron más de una noche.*

____

What reason(s)? [PA-NH Baseline]

*¿Por qué razones?*

__________________________________ ________________________________________

Which hospital(s)? Check all that apply. [PA-NH Baseline]

*¿Qué hospital? Marque todas las que apliquen.*

🞎 Yale-New Haven Hospital (YNHH)

🞎 YNHH Saint Raphael Campus (SRC)

🞎 Other (specify): ________________________________________

*Otro (especifique):* ________________________________________

During the past 12 months, have you visited a hospital Emergency Room (ER) for medical care? [NHS]

*En los últimos 12 meses, ¿ha usted visitado la sala de emergencia del hospital?*

🞎 Yes

*Sí*

🞎 No

*No*

If yes:

How many times did you visit a hospital Emergency Room (ER) for medical care during the past 12 months? [PA-NH Baseline]

*¿Cuántas veces, en los últimos 12 meses, ha usted visitado la sala de emergencia del hospital para atención medica?*

____

Which Emergency Room (s)? Check all that apply. [PA-NH Baseline]

*¿Qué sala de emergencia? Marque todas las que apliquen.*

🞎 Yale-New Haven Hospital (YNHH)

🞎 YNHH Saint Raphael Campus (SRC)

🞎 Other (specify): ________________________________________

*Otro (especifique):* ________________________________________

Tell me which of these apply to your Emergency Room (ER) visits in the previous year? [Adapted from NHIS]

*Dígame ¿cuál de estos puntos aplican a la razón por la cual usted visitó la sala de emergencia en el último año?*

|  | Yes  *Sí* | No  *No* | Don’t Know  *No sabe* |
| --- | --- | --- | --- |
| The problem was too serious for a doctor’s office or clinic  *El problema fue muy serio para una oficina de doctor o clínica* | ❑ | ❑ | ❑ |
| Only a hospital could help you  *Solo un hospital podía ayudarme* | ❑ | ❑ | ❑ |
| You didn’t have another place to go  *No tenía otro lugar donde ir* | ❑ | ❑ | ❑ |
| Your doctor’s office or clinic was not open  *La oficina de su doctor o clínica no estaba abierta* | ❑ | ❑ | ❑ |
| You could not reach your doctor or health care provider on the phone  *No pudo contactar a su doctor o proveedor de salud por teléfono* | ❑ | ❑ | ❑ |
| You could not get an appointment with your doctor or health care provider soon enough  *No pudo conseguir una cita oportuna con su doctor o proveedor de salud* | ❑ | ❑ | ❑ |
| Your health provider advised you to go (specify provider: ____________________________________________________________)  *Su proveedor de salud le recomendó que fuera (Especifique proveedor: ____________________________________________________________)* | ❑ | ❑ | ❑ |
| The Emergency Room is your closest provider  *La Sala de Emergencia es mi proveedor de salud más cercano* | ❑ | ❑ | ❑ |
| The Emergency Room is the most convenient place to get health care  *La Sala de Emergencia es el sitio más conveniente para recibir cuidados* | ❑ | ❑ | ❑ |
| The Emergency Room provides better care than you can get elsewhere  *En la Sala de Emergencia recibo mejor cuidado comparado a otro lugar* | ❑ | ❑ | ❑ |
| You get most of your care at the Emergency Room  *Recibo la mayoría de mi cuidado en la Sala de Emergencia* | ❑ | ❑ | ❑ |
| You arrived by ambulance or other emergency vehicle  *Llegó por ambulancia u otro vehículo de emergencia* | ❑ | ❑ | ❑ |

**HEALTHCARE ACCESS**

**Now I am going to ask about your overall ability to get and coordinate medical care:**

***Ayora le voy a preguntar sobre su capacidad general para obtener y coordinar servicios de salud:***

Overall, in the last 12 months, how easy or hard was it to get the medical care you needed? Include both primary and specialty care, medical tests, etc. [PA-NH Baseline]

*En los últimos 12 meses, ¿qué tan fácil o difícil le fue para conseguir la atención medica que usted necesita? Incluya atención primaria, atención especializada, o exámenes médicos, etc.*

🞎 Very easy

*Muy Fácil*

🞎 Somewhat easy

*Algo Fácil*

🞎 Somewhat hard

*Algo Difícil*

🞎 Very hard

*Muy Difícil*

In the last 12 months, were you unable to obtain medical care, tests, or treatments that you or a doctor believed necessary? [MEPS]

*En los últimos 12 meses, ¿no pudo obtener cuidado médico, exámenes, o tratamientos que usted o su doctor creía necesarios?*

🞎 Yes

*Sí*

🞎 No

*No*

In the last 12 months, did you delay obtaining medical care, tests, or treatments that you or a doctor believed necessary? [Adapted from MEPS]

*En los últimos 12 meses, ¿usted retrasó su cuidado médico, exámenes, o tratamientos que usted o su doctor creía necesarios?*

🞎 Yes

*Sí*

🞎 No

*No*

In the last 12 months, were you unable to obtain prescription medicines that you or a doctor believed necessary? [MEPS]

*En los últimos 12 meses, ¿no pudo obtener medicamentos recetados que usted o su doctor creía necesarios?*

🞎 Yes

*Sí*

🞎 No

*No*

In the last 12 months, did you delay obtaining prescription medicines that you or a doctor believed necessary? [Adapted from MEPS]

*En los últimos 12 meses, ¿usted se retrasó en obtener medicamentos recetados que usted o su doctor creía necesarios?*

🞎 Yes

*Sí*

🞎 No

*No*

How prepared are you to coordinate your own health care? [Community Care Coordination Patient Survey]

*¿Qué tan preparado está usted para coordinar su propio cuidado médico?*

🞎 Very prepared

*Muy Preparado*

🞎 Somewhat prepared

*Algo Preparado*

🞎 Mostly not prepared

*No Muy Preparado*

🞎 Not at all prepared

*Para Nada Preparado*

Having an illness often means doing different tasks and activities to manage your condition. How confident are you that you can do all the things necessary to manage your condition on a regular basis? [Stanford Patient Education Research Center Chronic Disease Self-Efficacy Scale]

*Tener una enfermedad aveces significa que tiene que manejar su condición con diferentes tratamientos o actividades. ¿Qué tan seguro está usted de que puede hacer todas las cosas necesarias para manejar su condición en un ritmo normal?*

Not at all confident 1 2 3 4 5 6 7 8 9 10 Totally confident

*Para Nada Seguro Completamente Seguro*

******END HERE FOR ED PILOT CONTROL PATIENTS******

**SATISFACTION WITH PA-NH**

**Now I am going to ask about your satisfaction with Project Access:**

***Ahora le voy a preguntar acerca de su satisfactión con Project Access:***

Did you receive medical care and/or services through PA-NH? Only include visits for medical care or services that the Project Access staff arranged for you. *[Non-ED patients only]*

*¿Recibió ayuda médica y/o servicios a travez de Project Access? Solo incluya las visitas médicas o servicios que un miembro de Project Access le hizo cita para usted.*

🞎 Yes

*Sí*

🞎 No

*No*

[If no, skip to end of survey]

In the past 12 months, when you made an appointment with a doctor or provider through Project Access, how often did you get an appointment as soon as you needed? [Adapted from CAHPS]

*En los últimos 12 meses, cuando usted hizo una cita con su médico o proveedor a travez de Project Access, ¿con que frecuencia consiguió usted la cita tan pronto como la necesitaba?*

🞎 Never

*Nunca*

🞎 Sometimes

*Algunas veces*

🞎 Usually

*Usualmente*

🞎 Always

*Siempre*

🞎 Have not made an appointment with a doctor or provider through Project Access in the past 12 months

*No he hecho una cita con mi médico o proveedor a travez de Project Access en los últimos 12 meses*

In the past 12 months, when you phoned Project Access with a medical question, how often did you get an answer to your medical question the same day? [Adapted from CAHPS]

*En los últimos 12 meses, cuando usted llamó a la oficina de Project Access con una pregunta médica, ¿con que frecuencia le respondieron el mismo día?*

🞎 Never

*Nunca*

🞎 Sometimes

*Algunas veces*

🞎 Usually

*Usualmente*

🞎 Always

*Siempre*

🞎 Have not phoned Project Access with a medical question in the past 12 months

*No he llamado a la oficina de Project Access con una pregunta médica durante el horario de atención*

In the past 12 months, how often did the Project Access staff treat you with courtesy and respect? [Adapted from CAPHS]

*En los ultimes 12 meses, ¿con que frecuencia los miembros de Project Access lo/la tratan con respeto y cortesía?*

🞎 Never

*Nunca*

🞎 Sometimes

*Algunas veces*

🞎 Usually

*Usualmente*

🞎 Always

*Siempre*

In the past 12 months, how often did the Project Access staff explain things in a way that was easy to understand? [Adapted from CAHPS/MEPS]

*En los últimos 12 meses, ¿con qué frecuencia los miembros Project Access le explicaron las cosas de una manera que fuera fácil de entender?*

🞎 Never

*Nunca*

🞎 Sometimes

*Algunas veces*

🞎 Usually

*Usualmente*

🞎 Always

*Siempre*

In the past 12 months, how often did the Project Access staff listen carefully to you? [Adapted from CAHPS/MEPS]

*En los últimos 12 meses, ¿con que frecuencia los miembros de Project Access lo/la escucharon con atención?*

🞎 Never

*Nunca*

🞎 Sometimes

*Algunas veces*

🞎 Usually

*Usualmente*

🞎 Always

*Siempre*

In the past 12 months, how often did the Project Access staff spend enough time with you? [Adapted from CAHPS/MEPS]

*En los últimos 12 meses, ¿con que frecuencia los miembros de Project Access se tomaron suficiente tiempo con usted?*

🞎 Never

*Nunca*

🞎 Sometimes

*Algunas veces*

🞎 Usually

*Usualmente*

🞎 Always

*Siempre*

How easy or difficult was it to get the medical care or services that you needed through Project Access?

*¿Qué tan fácil o difícil fue hacer las citas para ver a su doctor/especialista  que vió a travez de Project Access?*

🞎 Very easy

*Muy Fácil*

🞎 Somewhat easy

*Algo Fácil*

🞎 Somewhat hard

*Algo Difícil*

🞎 Very hard

*Muy Difícil*

How satisfied were you with the amount of time you waited between enrolling in Project Access and receiving the medical care or services you needed?

*¿Qué tan satisfecho está usted con el tiempo que tuvo que esperar entre el momento que ingreso al programa de Project Access y el recibir la atención médica o los servicios que usted necesitaba?*

🞎 Very satisfied

*Muy satisfecho*

🞎 Somewhat satisfied

*Algo satisfecho*

🞎 Somewhat dissatisfied

*Algo insatisfecho*

🞎 Very dissatisfied

*Muy insatisfecho*

How easy or difficult was it to follow the treatment advice given to you by the doctor(s) or health care provider(s) that you saw through Project Access? This includes getting additional tests, filling/taking medications, etc.

*¿Qué tan fácil o difícil fue seguir el tratamiento que fue dado por el médico/proveedor de salud que vió a travez de Project Access? Esto incluye el hacerse exámenes adicionales, comprando y tomando medicación, etc.*

🞎 Very easy

*Muy Fácil*

🞎 Somewhat easy

*Algo Fácil*

🞎 Somewhat hard

*Algo Difícil*

🞎 Very hard

*Muy Difícil*

How would you rate the care you received from the doctor(s) or health care provider(s) you saw through Project Access?

¿Qué puntuación le daría al servicio médico/doctor que vió a travez de Project Access?

🞎 Excellent

*Excelente*

🞎 Very good

*Muy Buena*

🞎 Good

*Buena*

🞎 Fair

*Regular*

🞎 Poor

*Mala*

While participating in Project Access, did any of the following issues or concerns impact your ability to get the health care services you need? Check all that apply. [Expanded from PA-NH Baseline]

*Cuando usted participaba en Project Access ¿Algunos de los siguientes puntos o consideraciones impactarón su habilidad para conseguir los servicios médicos que necesitaba? Marque todas las que apliquen.*

🞎 Financial concerns/cost (e.g., insurance premiums, sliding-scale fees, co-pays)

*Problemas Financieros/Costos*

🞎 Transportation

*Transportación*

🞎 Work schedule conflicts

*Conflicto con horas de trabajo*

🞎 Childcare

*Cuidado de niños*

🞎 Not sure how/where to get care

*No sabe cómo/donde conseguir cuidado*

🞎 Hard to find providers that take patients with no insurance or Medicaid

*Difícil encontrar proveedores que acepten pacientes sin seguro o con Medicaid*

🞎 Difficulty getting appointments soon enough

*Dificultad en conseguir una cita oportuna*

🞎 Difficulty communicating with providers (i.e., language barrier)

*Dificultad en comunicarse con proveedores (i.e. barrera de idioma)*

🞎 Difficulty understanding medical information

*Dificultad en entender información medica*

🞎 Difficulty filling prescription medications

*Dificultad en llenar medicamentos recetados*

🞎 Unhappy with past experience with a health care provider

*Insatisfecho con experiencias pasadas con proveedor de salud*

🞎 Prefer to treat myself

*Prefiero tratarse a si mismo*

🞎 Disability

*Discapacidad*

🞎 Other (specify): ______________________________

*Otro (especifique):* ______________________________

🞎 None

*Ninguno*

While you were participating in Project Access, would you say that your ability to get the medical care or services that you needed was…

*Cuándo usted participaba en Project Access, ¿usted diría que su habilidad de conseguir los cuidados médicos o servicios que usted necesitaba fue…*

🞎 Much better

*Mucho mejor*

🞎 Somewhat better

*Algo mejor*

🞎 About the Same

*Igual*

🞎 Somewhat worse

*Algo Peor*

🞎 Much worse

*Mucho peor*

How would you rate the services you received from the Project Access staff?

*¿Cómo calificaría al servicio que recibió a travez de los miembros de Project Access?*

🞎 Excellent

*Excelente*

🞎 Very good

*Muy Buena*

🞎 Good

*Buena*

🞎 Fair

*Regular*

🞎 Poor

*Mala*

How helpful was each of the following Project Access services?

*¿De cuanta ayuda fue Project Access en los siguientes servicios?*

🞎 Extremely helpful

*Extremadamente de ayuda*

🞎 Very helpful

*De mucha ayuda*

🞎 Somewhat helpful

*De algo de ayuda*

🞎 Slightly helpful

*De un poco de ayuda*

🞎 Not helpful at all

*De nada de ayuda*

- Assistance scheduling appointments

*Asistencia para hacer citas*

- Reminder calls before appointments

*Llamadas recordatorias antes de las citas*

- Follow up calls after appointments

*Llamadas despues de las citas*

- Having a Patient Navigator attend appointment(s) with you *[ED patients only]*

*El tener un Navegador de Pacientes que vaya a las citas con usted*

- Assistance with transportation

*Asistencia con la transportacion*

- Assistance understanding your medical care

*Asistencia para entender cuidados medicos*

- Assistance obtaining test results and reports

*Asistencia en obtener resultados y reportes*

- Assistance obtaining free or low-cost medications

*Asistencia en obtener medication gratis o de bajos costos*

- Referrals for other services (e.g., housing, food, financial assistance)

*Referidos a otros servicios (Ej. Vivienda, comida, asistencia financiera)*

- Having someone to talk to about your health/health care (e.g., emotional support)

*Tener a alguien para hablar de su salud/cuidados médicos (ej. Ayuda emocional)*

- Other (specify): ______________________________

*Otros (especifique):* ______________________________

Which one of the above Project Access services was most helpful?

*De los servicios mencionados anteriormente, ¿Cuál fue el de más ayuda?*

🞎 Assistance scheduling appointments

*Asistencia para hacer citas*

🞎 Reminder calls before appointments

*Llamadas recordatorias antes de las citas*

🞎 Follow up calls after appointments

*Llamadas despues de las citas*

🞎 Having a Patient Navigator attend appointment(s) with you *[ED patients only]*

*El tener un Navegador de Pacientes que vaya a las citas con usted*

🞎 Assistance with transportation

*Asistencia con la transportacion*

🞎 Assistance understanding your medical care

*Asistencia para entender cuidados medicos*

🞎 Assistance obtaining test results and reports

*Asistencia en obtener resultados y reportes*

🞎 Assistance obtaining free or low-cost medications

*Asistencia en obtener medication gratis o de bajos costos*

🞎 Referrals for other services (e.g., housing, food, financial assistance)

*Referidos a otros servicios (Ej. Vivienda, comida, asistencia financiera)*

🞎 Having someone to talk to about your health/health care (e.g., emotional support)

*Tener a alguien para hablar de su salud/cuidados médicos (ej. Ayuda emocional)*

🞎 Other (specify): ______________________________

*Otros (especifique):* ______________________________

🞎 None of the above

*Ninguno de los anteriores*

Are there services that Project Access did not provide that would have helped improve your ability to get the medical care you needed?

*¿Hay algun servicio que Project Access no proporcionó que hubiera sido de ayuda para mejorar su habilidad de conseguir los servicios médicos que usted necesitaba?*

_____________________________________________________________________________________

How satisfied or dissatisfied are you with your overall experience with Project Access?

*¿Qué satisfecho o insatisfecho esta con la experiencia general que tuvo con Project Access?*

🞎 Very satisfied

*Muy satisfecho*

🞎 Somewhat satisfied

*Algo satisfecho*

🞎 Somewhat dissatisfied

*Algo insatisfecho*

🞎 Very dissatisfied

*Muy insatisfecho*

On a scale of 0-10, how likely is it that you would recommend Project Access to a friend or family member who needed help obtaining medical care or services?

*En la escala del 0 al 10, ¿cual es la probabilidad de que usted recomiende Project Access a un amigo o a un miembro de su familia que necesite ayuda para obtener ayuda y servicios médicos y servicios?*

🞎 0 🞎 1 🞎 2 🞎 3 🞎 4 🞎 5 🞎 6 🞎 7 🞎 8 🞎 9 🞎 10
