# Supplementary material for: “I wanted to participate in my own care”: Evaluation of a Patient Navigation Program
Source: West J Emerg Med. 2021 Feb 22;22(2):417–26. doi: 10.5811/westjem.2020.9.48105 (PMC7972383; doi:10.5811/westjem.2020.9.48105)
Supplement: Supplementary file 2 [file wjem-22-417-s002.doc]

**Project Access-New Haven Emergency Department Frequent User Pilot**

**Semi-Structured Qualitative Interview Guide**

Good morning/afternoon, my name is _______. I am a doctor hoping to understand the barriers associated with accessing health care, your views on primary care and emergency room use, the services you received though Project Access-New Haven (a program you were a part of), things that you found helpful about the program, and things they could improve upon. Before we get started, we will go over some additional information about the interview and you will have a chance to ask questions. After you have all of the information, you can decide whether to go ahead with the interview.

INTERVIEWER NOTE: *Review HIC-approved information sheet prior to beginning interview. If patient agrees to participate, sign the last page of the information sheet and proceed. Provide the patient with a copy of the information sheet at the interview (if interview is in person) or via mail (if interview is via phone).*

If you have no further questions, I will turn on the recorder now:

1. What part of New Haven are you from? How long have you lived here? (Ice breaker).
2. Before you enrolled in the program, can you tell me a little bit about how you used the emergency room?

Probe: reasons for ED care, transportation issues, care coordination issues, where do you go when you are sick.

2a. Before you enrolled, how did you use primary care services?

1. Can you tell me about Project Access-New Haven and their patient navigation program?

Probes: what is the program, what did they do for you, how did they help you, what do you believe was the goal of the program, what did you learn from the program, etc.

1. What were some of things the program did for you that were most helpful? I would like you to think back to the past and try to remember the best thing that happened to you from being a part of the program. Would you be willing to share that with me?

Probes: was there a time when you needed social help, medical help, transportation, having someone to talk to you, easily access care.

1. Specifically, how did you feel about the patient navigator accompanying you to your first appointment?

Probes: did you want more time/more appointments together, how was it, good or not good, did you feel you got something out of it?

1. I would like to ask you about a bad experience you had while being in the program. Would you be willing share that with me?
2. What are some things that you believe could be done better?
3. Can you tell me what do you do now when you get sick?

Probe: do you call your doctor, do you go to the ED, was this something that you did before or something the program helped you with.

1. Can you tell me how you used the ED after you enrolled in the program?

Probes: do you triage when to go to the ED, do you contact your doctor first, and do you ask questions during your doctor’s visit that you didn’t before, any changes in how they approach using the ED.

1. How did you use the primary care clinics/provider before using the program? How about after being done with the program?

Probe: has your perception of your primary care doctors/providers changed.

1. You had multiple navigators (Juan and Adrienne) helping you, and also other people involved in your care, while you were involved in the Project Access program. I am wondering if you could tell me a little bit about everyone and how each person helped you.

Probe: how was beneficial was the bridge of the navigators between you and your doctor, did it close a gap or was it more work or required more steps in your connecting with your doctor.

1. Was there a health care need that was not met with the program or that you would like to get addressed?

Probes: specialty access (did you find it harder or easier to make appointments), mental health access, doctors that don’t take their insurance, resources for doctors that will see patients.

1. If you had the chance to re-design the program, how would you do it?

Probe: have it for longer, shorter, more reminders, more follow up, etc.

1. Now that you are done with program, how will you use primary care and the ED? What will you do?
2. Overall, would you recommend this program to one of your friends or family members?

Thank you so much for your time, I would like to share my card with you and see if you have anything you would like to tell me. Would it be okay for me to contact you tomorrow by phone?
