# Supplementary material for: “I wanted to participate in my own care”: Evaluation of a Patient Navigation Program
Source: West J Emerg Med. 2021 Feb 22;22(2):417–26. doi: 10.5811/westjem.2020.9.48105 (PMC7972383; doi:10.5811/westjem.2020.9.48105)
Supplement: Supplementary file 3 [file wjem-22-417-s003.docx]

| **Table 4:** Interview Themes and Subthemes. | |
| --- | --- |
| **Theme and sub theme** | **Illustrative Quotes** |
| **Theme 1: Patient navigators were perceived as effective health care coordinators and patient advocates who provided continuity and individualized support** | |
| *Navigator program provided support for those without strong health care networks* | “[The PN was] really helpful, my son has autism and it’s hard to find a pediatric doctor that specializes in autism that takes our insurance. He actually went in and found some information out for me about doctors around [the city] and the surrounding areas that take my insurance and specialize with children in autism or children with all different disorders so, yes, it was very helpful” (Participant 6). |
| *Navigators were viewed as reliable, trustworthy, non-judgmental people that could be depended on for support and help.* | “That’s the reason you're working with them because you feel like nobody elsewhere is helping you and they’re there to help… I was at my wit’s end when [the PN] came to me. I was so fed up. I was so tired like cussing. I was in the hospital cussing everybody out. [*laughter*] I was because I'm just tired feeling like I'm not getting any help. I'm not being heard. I'm not being treated properly” (Participant 5).  “And what I love, you know, they didn’t treat me like I was just anybody, they treated me like somebody important, no matter what my insurance was. That was very, very, very helpful for me. And it does help when you’re down and out and you got somebody on your side that’s not nasty and don’t treat you like you’re nothing because you don’t have the funds or means to pay for the higher price of insurance, and you have the state’s insurance. It’s important that people treat you the same” (Interview 8).  “[The PNs are] there to help you. Motivate you to go to your programs, you know, to your doctor’s appointments. And if you didn’t have bus fare to get there, they make sure you get there but the transportation if you have a medical card. I always told them my business and they were always there to listen.” (Participant 10). |
| *Navigator support fostered feelings of self-worth and helped participants re-prioritize personal health.* | “[The PN] made me feel like someone was there to help me… he was there and he sat there and he was saying feel free, you should ask questions. He made me, he helped me realize you’re paying for this, you have the right to ask questions, you have the right to tell them, ‘you know what I need this kind of pain medicine, this doesn’t work for me,’ and that helped me out a lot” (Participant 8). |
| **Theme 2: Navigators helped patients address health related social needs that were drivers and barriers to health care utilization.** | |
| *Participants frequently encountered issues with transportation, housing, insurance, or employment that impacted healthcare utilization.* | “I had fallen behind on my care, because I have three children and I have one that has a disability. [The intervention] helped me out a lot. It was just that, that little reminder of, ‘Hey you have that appointment coming up on Thursday. Do you want to still keep it? How is your weekend going? How are you doing with childcare, transportation?’ You know it was, it was comforting. It was really comforting. ‘And at times I got a phone call and I’m like, I have an appointment this morning?’ It just felt good to have that extra support for a single mom like me of three children” (Participant 6). |
| *Navigators were noted to go above and beyond program expectations to help patients address social factors that impacted patient health and healthcare utilization* | “[The PN] was really helpful. She actually even helped me with a couple of other things that didn’t have to with the primary care physician. I went through some medical illnesses. I went through some problems within my own personal life. She went to find the proper precautions as far as staffing, medical offices. Like she really helped me and called me and gave me numbers to different places. She was definitely very helpful. I probably wouldn’t haven’t stayed with the Project Access if it wasn’t for [the PN]” (Participant 5). |
| **Theme 3: Primary care utilization was driven by clinic accessibility and quality of interpersonal relationships.** | |
| *Presence or absence of trust, continuity, and strong interpersonal relationship with a primary care provider influenced decisions to utilize primary care* | “I feel like it wasn’t that personal, like it wasn’t nice. [The primary care center] were just – just get right to the point and go. Then long waits but then rushed to get out the door. … the OB/GYN, she knows me because she’s been my doctor a long time. So, you don’t know a doctor, you don’t feel comfortable telling them things, but you have to for your health and then they just, ‘Mm-hmm, mm-hmm, mm-hmm. Yes, yes, yes. Okay. We’re going to give you this. Okay. Have a nice day’” (Participant 1).  “She knew everything about me because she read my chart, and how I know she read my chart, she knew what surgeries I had, the medications I was on, before she even talked to me. I didn’t have to do any explaining of what was going on with me right then and there. And that made me feel like you are taking an interest in me” (Participant 8).  “My primary care provider really listens to you and makes sure that he goes into details about what's going on with you and if you call him, he will call you right back. It's not like most doctors.” (Participant 9). |
| *Positive and negative interactions with staff impacted participants’ perceptions of care quality and accessibility, and decisions to seek care at the primary care office vs. emergency department.* | “When you go [to the Primary Care Center], [the staff] have attitudes. They seem like they don’t want to be working…When you try to make appointment, they expect everything to work for you at that point. For example - let's just say – ‘Does September 12 work for you?’ ‘Actually no, it doesn’t. I have another appointment.’ ‘Huh!’ Like just because it’s available on your booking system, doesn’t mean it’s available to me. They expect everything to go their way. They don’t accommodate customer service at all” (Participant 5).  “Well, with the primary care clinic, is for one they are overpopulated. They, they’re not able to assess each patient the way that they should.” (Participant 6). |
| *Appointment availability, time to appointment, and time with provider during appointment were key factors in choosing to utilize emergency care instead of primary care* | *“*It’s not that I couldn’t make the appointments. It’s if I made an appointment it would be weeks down the road and [my daughter] would’ve been better by then” (Participant 3 *describing why she used the ED for her daughter*).  “... when I tried to call the Primary Care Center, they weren’t available the way I needed them to be available... if I felt there was something important and medically urgent and to them it wasn’t, I wanted it that same day and they would do three, four days later and I felt to myself it was important, I would just go straight to the ED” (Participant 5).  “It’s like [the Primary Care Center providers] don’t really know your history. They just know my piece of paper. They bounce you around to so many different people. They have way too many patients. They can’t accommodate properly. You don’t want to keep seeing different people. You want to see the same person. Sometimes, when I go there, I'm rushed. You're always bounced around to different people where you’d have to explain your whole story to because they don’t know you. So there goes your 15 minutes right there. You got to re-explain everything and by the time, they’ve done explaining, they have only like a couple of minutes to assess you and then think what’s going on. That’s not the way it should work.” (Participant 6). |
| **Theme 4: Emergency Department provided convenient, comprehensive care for urgent needs and filled gaps in primary care access** | |
| *Utilization was driven by urgent care needs, convenience, and lack of access or satisfaction with services at primary care* | “I have to go to the emergency room because they’re the only ones who could give me something to calm me down. It doesn’t take [the pain] away but it will calm me down” (Participant 2).  “Because I felt as though when I tried to call the primary care center, they weren’t available the way I needed them to be available as for example if I felt something was to me - if I felt there was something important and medically urgent and to them it wasn’t, I wanted it that same day and they would do three-four days later and I felt to myself it was important, I would just go straight to the ED” (Participant 6). |
| *Despite long wait times and lack of provider continuity, emergency care was perceived as comprehensive and thorough* | “I love [the ED]. You get treated the way you should get treated at a doctor’s appointment. Properly assessing, hearing out what’s wrong. I like the way they check everything they needed. So, when I feel I got to go the ED because it’s the ED, they’d have to do all those necessary precautions because it’s an emergency department. The doctors come talk to you, really check it out, take care what you got to do and then you leave the way you wanted to be treated at a primary care center which they don’t do that. The ED was my first choice” (Participant 5) |

*ED*, emergency department; *PN,* patient navigation.
